# The Effects of Ceftriaxone on Glutamate Transporter Expression and the Gut Microbiome: Implications for a Role of Antibiotic-Induced Dysbiosis in Mediating Drug Seeking and Relapse

**DOI:** 10.1080/29933935.2024.2393727

**Published:** 2024-09-27

**Authors:** Andrew D. Winters, Dina M. Francescutti, David J. Kracht, Donald M. Kuhn, Mariana Angoa-Perez

**Affiliations:** John D. Dingell VA Medical Center and Department of Psychiatry & Behavioral Neurosciences, Wayne State University School of Medicine, Detroit, MI, USA

**Keywords:** Ceftriaxone, glutamate transporter, gut micobiota, relapse

## Abstract

Ceftriaxone (CTX), a beta-lactam antibiotic, is widely used in drug seeking and relapse studies due to its ability to enhance glutamate transporter (GLT-1) expression in the brain. Since increased synaptic glutamate is believed to drive drug seeking and relapse, CTX’s effect on GLT-1 offers potential for treating substance abuse. However, the effect of CTX on the gut microbiome remains unexplored. Mice received CTX at 200 mg/kg per day for 4 d, and its effects on the gut microbiome were assessed. CTX led to increased striatal GLT-1 expression and induced rapid, long-lasting dysbiosis, with females showing a greater response than males. Diversity metrics were significantly altered during the acute phase of CTX treatment. Alpha diversity showed varying recovery levels depending on sex, while beta diversity indicated that CTX-treated mice remained significantly different from controls. CTX caused significant increases in Bacillota and reductions in Bacteroidota. Most taxa were rapidly reduced by CTX, but *Enterococcus* and Bacillales expanded significantly. Metabolomic analysis revealed significant changes in microbial pathways related to substance use disorders. These findings indicate that CTX causes immediate and persistent alterations in the gut microbiome, highlighting the importance of considering the gut microbiome as a target in substance abuse treatment.

## Introduction

Ceftriaxone (CTX) is a third-generation cephalosporin antibiotic within the larger class of beta-lactam antibiotics. CTX emerged as a valuable tool in neurobiology research when it was discovered to possess neuroprotective effects by increasing glutamate transporter (GLT-1) expression.^1^ Increases in GLT-1 would serve to decrease excitatory neurotransmission by removing glutamate from the synapse. Soon after this discovery by Rothstein et al.,^1^ investigations into substance use disorders (SUD) incorporated CTX to study the hypothesis that excessive synaptic glutamate was driving relapse to drug use, which could be overcome by CTX-induced increases in GLT-1 expression. However, the majority of studies into CTX–drug abuse interactions did not account for the possibility that CTX, as a beta-lactam antibiotic, was exerting at least some of its effects on drug seeking and relapse via alterations in the gut microbiome.^2^

CTX has been used effectively in gut microbiome research for its dysbiotic effects and as a model compound for investigating gut recolonization, dissemination of selected bacterial taxa, and amplification of antimicrobial resistance after antibiotic disruption.^3–6^ CTX causes significant alterations in the gut microbiomes of animals^7–11^ and humans.^12,13^ However, microbiological studies of CTX generally use doses and durations of treatment that are very different from experiments investigating neuroprotection and prevention of drug relapse. For instance, studies on the gut employ doses of CTX ranging from 2000 to 4000 mg/kg and treatment times of 7 d to 11 weeks.^7,8,14^ On the other hand, neuroprotection and SUD studies use a treatment regimen of 200 mg/kg per day for four consecutive days based on the method of Rothstein et al.,^1^ as reviewed by Angoa-Perez and Kuhn.^2^ Because of the higher doses and longer treatment times used in microbiological studies of CTX and in consideration of the fact that studies showing its effectiveness in preventing drug seeking and relapse did not assess the status of the gut microbiome after CTX treatment, the present study was carried out to determine the extent to which this beta-lactam antibiotic modifies the gut microbiome. The results show that CTX significantly increases GLT-1 expression and coincidentally causes a rapid-onset and long-lasting alteration in the diversity and structure of the gut microbiome, opening the door for the possibility that microbiome dysbiosis could play a role in CTX-induced reductions in drug seeking and relapse, particularly in relation to sex differences.

## Materials and methods

### Animals

A total of 78 C57BL/6 mice (39 females, 39 males) from Charles River were used in this study. The mice were 7–8 weeks of age with body weights of 20–22 g for males and 15–18 g for females at the start of the experiment. All mice were given *ad libitum* access to water and normal rodent laboratory chow (LabDiet PicoLab Rodent Diet 5LOD). Mice were housed in a room with constant temperature and humidity and with alternating 12-hour periods of light and darkness. Stressors such as noise and handling by multiple persons were avoided and mice were monitored daily for signs of distress or injury until the experimental endpoints. The Institutional Care and Use Committee of Wayne State University approved the animal care and experimental procedures (IACUC 22-02-4406). All procedures were also in compliance with the NIH Guide for the Care and Use of Laboratory Animals and were conducted according to ARRIVE guidelines.

### CTX treatment

Mice were randomly assigned to a control group (*N* = 36) or a CTX treatment group (*N* = 42). Six independent cohorts of mice (*N* = 13 per cohort) were used, and each experiment was comprised of the two treatment groups, all from the same cohort. Mice from the same Treatment group were housed 2–4 per cage. CTX was prepared in sterile water and was administered ip at a dose of 200 mg/kg once per day for 4 consecutive days, essentially following protocols used by the majority of studies that examined CTX effects on drug abuse and relapse (see^2^ for review). Time-matched controls received ip injections of sterile water. Injections were given in a volume of 10 ml/kg using Becton Dickinson ½ ml syringe with permanently attached 27 g × ½ in needle. Body weights were recorded for all mice during CTX treatment. For collection of fecal pellets, mice were moved singly into a sterile cage without bedding and fecal pellets were collected using sterile forceps and placed into sterile tubes immediately prior to the first treatment with CTX (Day 1), 24 h after the first treatment (Day 2), 24 h after the last CTX treatment (referred to hereafter as Day 5), and thereafter on Day 8, Day 12, Day 65, and Day 100. This time frame was chosen to allow analysis of CTX effects at the acute times used in most drug abuse studies (Day 5–Day 12) and at extended times (Day65–D100) to monitor recovery from CTX treatment. Fecal samples were subjected to quantitative PCR (qPCR) analyses and 16S rRNA gene sequencing (see below). For immunoblotting analysis, 4 cohorts composed of 24 controls (12 males, 12 females) and 28 CTX-treated mice (14 males, 14 females) were sacrificed on D5 by decapitation. The striatum was isolated from brains for GLT-1 expression evaluations. The cecum was also removed from these same mice on Day 2 and Day 5 and weighed.

### Determination of bacterial load

Bacterial load, defined here as bacterial 16S rRNA gene copy number per mg of feces, was assessed via amplification of the V4 region of the 16S rRNA gene using qPCR. Each 20-µl reaction consisted of 10 µL of PowerUp™ SYBR™ Green Master Mix (Applied Biosystems, Waltham, MA), 0.8 µM of 515F (5’-GTGCCAGCMGCCGCGGTAA-3’) and 806 R (5’-GGACTACHVGGGTWTCTAAT-3’) primers (Integrative DNA Technologies, Coralville, IA), 6.68 µL nuclease free water, and 3 µL of purified DNA or water. Each qPCR run was performed using the following parameters: 95°C for 2 min, followed by 37 cycles of 95°C for 30 s, 57°C for 30 s, and 72°C for 30 s. All qPCRs and data collection were carried out with an ABI QuantStudio 3™ real-time PCR system using software v2.3 (Applied Biosystems, Waltham, MA, USA). Raw amplification data were analyzed using the online platform Thermo Fisher Cloud, Standard Curve Analysis Module online platform (version 3.9) with an automatic baseline setting and a fluorescence threshold of 0.25. Cycle of quantification (Cq) values were calculated using the average cycle number required to produce exponential increases in normalized fluorescence. To assess 16S rRNA gene copy number, a gene block (gBlock®; Integrated DNA Technologies) was synthesized and used as a quantitation standard. The gene including protective bases (TGGCCA) flanking the target sequence (GenBank accession CP117235: position 476,730 to 477,021) had a length of 304 bp and a molecular weight of 292,744.1 g/mole. The gene block was rehydrated with 50 μl 1X Tris-EDTA buffer (10 mM Tris HCl, 1 mM disodium EDTA, pH 7.4; Fisher BioReagents, Pittsburgh, PA, USA) and quantified by using a Qubit 4.0 fluorometer (Thermo Fisher Scientific, Waltham, MA, USA). For all tests, the gene block was serially diluted in 100 ng/μl tRNA (Carrier RNA; Qiagen, Carlsbad, CA, USA) in RNase-free water (Fisher BioReagents). Standard curves were included on each qPCR run in duplicate, and a linear regression of the 16S rRNA gene copy number and Cq value was plotted from each standard curve. Copy number of the 16S rRNA gene in samples was then calculated as described by Gallup^15^ using the equation Xσ = EAMPb-Cq. EAMP represents the exponential amplification value for the assay, which is represented as EAMP = 10-1/m, while m and b represent the slope and intercept of the above regression, respectively. DNA copy numbers were log_10_ transformed to normalize the data before analysis. The rstatix package version 0.7.2^16^ was used to detect outliers.

### 16S rRNA gene amplicon sequencing and bioinformatic processing

The V4 region of the 16S rRNA gene was amplified and sequenced via the dual indexing strategy developed by Kozich et al.^17^ as previously employed in our laboratory.^18,19^ 16S rRNA gene sequences were clustered into amplicon sequence variants (ASVs) using the Divisive Amplicon Denoising Algorithm (DADA2;^20^) pipeline to obtain merged, denoised, chimera-free, inferred ASVs as previously reported by our laboratory,^18,19^ with the exception that forward and reverse reads were truncated at 200 and 155 bases, respectively. The R package decontam version 1.20.0^21^ was used to identify ASVs that were likely potential background DNA contaminants based on their distribution among fecal samples and negative controls using the “IsContaminant” method. We determined an ASV to be a contaminant if it had a decontam P score ≥ 0.5 and was present in 75% of negative controls with an overall average relative abundance of at least 1.0% and a greater average relative abundance in controls compared to fecal samples. Based on these criteria, 11 ASVs were identified as DNA contaminants and were removed from the data set. Prior to the removal of any contaminant ASVs, the dataset contained a total of 8,572,911 sequences and 1,514 ASVs. After the removal of the 11 ASVs deemed to be potential DNA contaminants, 99.989% of sequences and 1,503 ASVs remained in the dataset. All samples had a Good’s coverage score of ≥98.6%.

### Immunoblotting

The effects of CTX treatment on striatum levels of GLT-1 were determined by immunoblotting. In short, striatum was dissected from the brain and stored at −80°C. Frozen tissue was sonicated in 1% SDS at 95°C, and insoluble material was removed by centrifugation. Soluble protein concentrations were determined by the bicinchoninic acid method. Equal amounts of protein were resolved by SDS-polyacrylamide gel electrophoresis and then electroblotted to nitrocellulose. Blots were blocked in Odyssey Intercept Blocking Buffer (LiCor Biosciences #927-60001) for 1 h at room temperature. Primary antibodies against GLT-1 (Abcam #106289, 1:500) or GAPDH (Sigma #G8795, 1:10,000) were added and incubated overnight at 4°C. Blots were washed in Tris-buffered saline + 0.01% Tween-20 and then incubated with IRDye secondary antibodies (LiCor Biosciences #926-32211 & #926-68070, 1:10,000) for 1 h at room temperature. Immunoreactive bands were visualized by enhanced fluorescence, and the relative densities of GLT-1 and GAPDH-reactive bands were determined by imaging with an Odyssey CLx Infrared Image System (LiCor Biosciences, Lincoln, NE) and quantified using Image Studio software (LiCor). GLT-1 relative densities were normalized to the GAPDH level for each lane to control for loading errors.

### Data analysis and statistics

Quantitative PCR (qPCR) was employed to evaluate log-transformed 16S rRNA gene copies across experimental conditions using a linear mixed-effects regression model (lmer) implemented with the lme4 package in R.^22^ The model included Treatment group, Day, and Sex as fixed effects, with Mouse_ID as a random effect to account for within-subject variability. ANOVA (car package)^23^ was used to determine statistical significance among factors, and Tukey’s method (emmeans package)^24^ was applied for pairwise comparisons to identify significant differences between experimental groups.

GLT-1 expression levels, body weight variations, and cecum weight were analyzed using ANOVA and Tukey’s post hoc tests (emmeans package)^24^ for pairwise comparisons. For GLT-1 expression, two ANOVA models (glm) were constructed: one examining main effects of Treatment group and Sex, and another assessing their interaction. Similarly, analyses of body weight and cecum weight employed two-way ANOVA models (glm) to investigate the main effects and interactions of Treatment group and Sex. Tukey’s method was subsequently applied to discern significant differences among experimental conditions (emmeans package).^24^

Prior to the analyses of alpha diversity metrics, the 16S rRNA gene profiles were subsampled to a sequencing depth of 8,363 reads per sample using Mothur software version 1.44.1,^25^ which resulted in the removal of five CTX samples with low read numbers from the dataset. Alpha diversity was characterized using Chao1 (i.e., richness), Shannon (i.e., heterogeneity) and Inverse Simpson (i.e., evenness) indices. Alpha diversity metrics were calculated using Mothur and assessed using a linear mixed-effects regression model (lmer). The model included Treatment group, Day, and Sex as fixed effects, with Mouse_ID as a random effect to account for within-subject variability. ANOVA (car package) was utilized to evaluate each diversity metric for statistical significance, followed by Tukey’s pairwise comparisons (emmeans package) to detect significant differences between experimental groups. For all analyses, fixed effects were assessed using Type II Wald F tests, and interactions were assessed using Type II Wald F tests.

Variation in the bacterial profiles of fecal samples from different treatment groups were visualized through Principal Coordinates Analyses (PCoA) using the R package vegan version 2.6.4.^26^ Statistical comparisons of bacterial community structure between CTX treatment groups and time-matched controls were made through permutational multivariate analysis of variance (PERMANOVA) using the R package vegan. Beta diversity was characterized using the Bray-Curtis dissimilarity index, for which values were calculated using percent relative abundance data for ASVs within samples.

ASVs with either an identical “Rank2” (e.g., Phylum) or “Rank6” (e.g., Genus) taxonomy were bioinformatically merged using the “tax_glom” function in the R package phyloseq version 1.44.0.^27^ Subsequently, the data underwent scaling via the trimmed mean of M-values method.^28^ All analyses involved control of the false discovery rate at a 1.0% level (*q* = 0.01). Assessment of correlations between striatal GLT-1 expression and bacterial ASVs was performed using MaAsLin2 with a false discovery rate at a 5.0% level (q = 0.05).

Differential abundance testing was performed on count data using the R package MaAsLin2 version 1.14.1.^27^ Negative binomial regression was employed within Maaslin2 to identify taxa associated with experimental conditions. The models included fixed effects for Treatment group, Sex, and GLT-1 expression, and all possible interactions. Prior to analysis, ASVs with identical taxonomic classifications at either “Rank2” (e.g., Phylum) or “Rank6” (e.g., Genus) were merged using the “tax_glom” function from the R package phyloseq version 1.44.0.^28^ The merged data underwent normalization and scaling via the trimmed mean of M-values (TMM) method.^29^ To control for multiple testing, all analyses involved controlling the false discovery rate (FDR) at a level of 1.0% (q = 0.01), except for correlations between striatal GLT-1 expression and bacterial ASVs, which used a 5.0% FDR threshold of 5.0% (q = 0.05). For analyses involving repeated measures, Mouse_ID was included as a random effect in these models to account for potential correlations due to repeated measures from the same subjects.

### Inference of functional genes and pathways

Phylogenetic Investigation of Communities by Reconstruction of Unobserved States (PICRUSt2) software package version 2.5.2^29^ was used for predicting functional pathway occurrence based on marker gene sequences (16S rRNA sequencing data). MetaCyc ontology predictions^30^ were used for metabolic pathway classification. Functional pathways were annotated based on the individual MetaCyc Superpathways.

## Results

### Pharmacological effects of CTX

**Figure 1.**
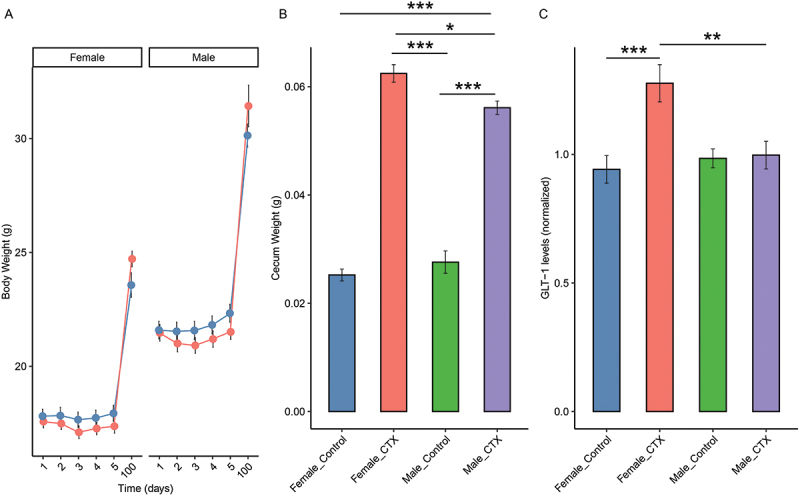
Pharmacological effects of ceftriaxone (CTX) treatment. (A) Body weights of mice treated with CTX compared to controls. (B) Cecum weights of ctx-treated mice compared to controls on D5. (C) Striatal GLT-1 protein levels of ctx-treated mice compared to controls on D5. Data are presented as mean ± standard error of the mean. *p < 0.05, **p < 0.01, and ***p < 0.001 vs. respective controls.

Three possible pharmacological effects of CTX were assessed during the treatment regimen: body weight, cecum weight, and striatal GLT-1 expression (Figure 1). Two mixed-effects models were employed to assess the factors influencing weight measurements in the experimental mice, with Mouse ID included as a random effect to account for individual variability (Figure 1A). Model 1 used Type II Wald F tests, revealing significant effects for Day (*F* = 914.55, d*F* = 5, *p* < 0.001) and Sex (*F* = 145.75, d*F* = 1, *p* < 0.001), suggesting that body weight varies significantly across different measurement days and between male and female mice. However, the effect of Treatment was not significant (*F* = 1.43, d*F* = 1, *p* = 0.236).

Model 2, analyzed with Type III Wald F tests, provided additional insights by including interaction terms. The significant interaction between Treatment and Day (*F* = 6.96, d*F* = 5, *p* < 0.001) indicated that body weight varied over time and was treatment-dependent. Post hoc comparisons further elucidated this interaction: during the CTX administration period (Days 2 to 5), there were no significant differences in body weights between the CTX and control groups except on Day 5, where control mice had significantly higher weights than the CTX group (*p* = 0.0491). By Day 100, the CTX group exhibited significantly lower weights than the control group (*p* = 0.0034). These results show that while CTX treatment did not substantially alter body weight during the administration period (D2-D5), a pronounced and sustained weight reduction became evident by Day 100 (Figure 1A).

The significant interaction between Day and Sex (*F* = 16.75, d*F* = 5, *p* < 0.001) highlighted differing body weight trajectories between male and female mice over time. Post hoc analyses revealed significant weight differences across all days compared to Day 100 within both sexes (all *p* < 0.0001), indicating progressive weight changes over the study period. In females, significant differences were observed between early days (Day 1 to Day 5) and Day 100, with weight consistently decreasing (all *p* < 0.0001). Similarly, for males, significant weight reductions were noted when comparing early days to Day 100 (all *p* < 0.0001). These patterns indicate a significant, progressive weight loss over time, likely reflecting the cumulative effect of experimental factors (Figure 1A).

Analysis of cecum weight, controlling for Treatment group and Sex, revealed a significant main effect of Treatment (F(1,22) = 289.8684, *p* < 0.001), showing substantial increases in cecum weight between subjects who received CTX and those in the control group (Figure 1B). Additionally, an interaction effect between Treatment and Sex was observed (F(1,22) = 7.9483, *p = *0.009988), indicating that the impact of treatment on cecum weight varied according to the sex of the subjects. However, the main effect of Sex alone did not reach statistical significance (F(1,22) = 1.0949, *p = *0.306750).

Further analyses through Tukey’s post-hoc tests revealed that CTX-treated females exhibited significantly higher cecum weights compared to control females (mean difference = 0.0372, *p* < 0.001). There was no significant difference in cecum weight between control males and females (mean difference = 0.0024, *p = *0.725). Comparisons between CTX-treated males and females showed a slight but significant difference in cecum weight (mean difference = −0.0063, *p = *0.030), with CTX-treated males exhibiting higher cecum weights compared to control males (mean difference = 0.0285, *p* < 0.001) (Figure 1B). Cecum weights were also significantly increased by CTX on D2 (Supplementary Figure S1).

Significant effects on striatal GLT-1 expression were observed for the Treatment and Sex factors, as well as their interaction (F(1,44) = 8.05, *p = *0.0068). Specifically, there was a significant main effect of the Treatment (F(1,44) = 16.80, *p = *0.00018), with CTX-treated animals exhibiting higher GLT-1 expression compared to control animals (coef = 0.163, *p* = 0.0062) (Figure 1C). Although the main effect of Sex was not significant (F(1,44) = 0.26, *p* = 0.6112), males showed lower GLT-1 expression compared to females (coef = 0.131, *p* = 0.0251).

Post-hoc Tukey tests within the interaction effect revealed that CTX-treated females exhibited a 0.335 higher GLT-1 expression compared to control females (*p* = 0.00098). In contrast, CTX-treated males showed lower GLT-1 expression compared to CTX-treated females (coef = 0.280, *p* = 0.00395), while there was no significant difference between CTX-treated and control males (*p >* 0.05) (Figure 1C). These findings indicate that both treatment with CTX and Sex significantly influence striatal GLT-1 expression, with CTX treatment particularly affecting females more than males.

### Effects of CTX on bacterial load

**Figure 2.**
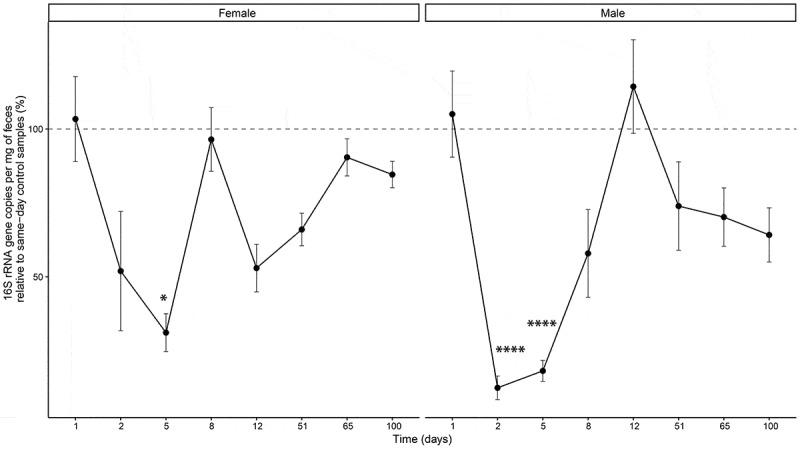
16S rRNA gene copies per mg of feces of female and male mice administered ceftriaxone (CTX) relative to time-matched control samples. Data are presented as mean ± standard error of the mean. *p < 0.05, ****p < 0.0001 vs. respective controls. CTX was administered 24 hours before sample collection on both Day 2 and Day 5.

Two mixed-effects models were employed to assess the factors influencing 16S rRNA gene copies. Model 1 used Type II Wald F tests, revealing significant effects for Treatment (*F* = 30.06, *p* = 2.4e-06), Day (*F* = 11.92, *p* = 2.5e-11), and Sex (*F* = 7.38, *p* = 0.0096). These findings indicate independent influences on 16S rRNA gene copy number by each factor. In contrast, Model 2, analyzed with Type III Wald F tests, showed no statistically significant interactions among Treatment group, Day, and Sex (all *p* > 0.05). This suggests that while individual factors significantly affect 16S rRNA gene copy number, their combined effects through interactions did not significantly influence the outcome measure in this study (Figure 2).

On Day 1, baseline levels for both males (105.04 ± 14.57% SE) and females (103.37 ± 14.37% SE) were similar relative to time-matched controls. By Day 2, there was a significant reduction in bacterial load in both males (12.46 ± 3.98% SE) and females (51.94 ± 20.20% SE) administered CTX. This reduction persisted on Day 5 for males (18.17 ± 3.54% SE) and females (31.12 ± 6.40% SE), though the magnitude varied slightly (Figure 2).

Recovery of 16S rRNA gene copies was observed by Day 8, with values approaching or exceeding baseline levels in both sexes (female: 96.47 ± 10.79% SE, male: 57.92 ± 14.87% SE). By Day 12, males (114.35 ± 15.83% SE) showed an increase beyond baseline, while females (52.96 ± 8.06 SE) displayed partial recovery. By Day 65 and Day 100, 16S rRNA gene copies remained comparable to baseline in males (Day 65: 70.21 ± 9.88% SE, Day 100: 64.17 ± 9.16% SE) and females (Day 65: 90.41 ± 6.28% SE, Day 100: 84.59 ± 4.47% SE) (Figure 2).

Furthermore, sex-specific differences were observed within the CTX treatment group. Specifically, on Day 2, females administered CTX exhibited significantly higher levels of 16S rRNA gene copies compared to males administered CTX (estimate = 0.756, *p* = 0.0189).

### Effects of CTX on bacterial community diversity

**Figure 3.**
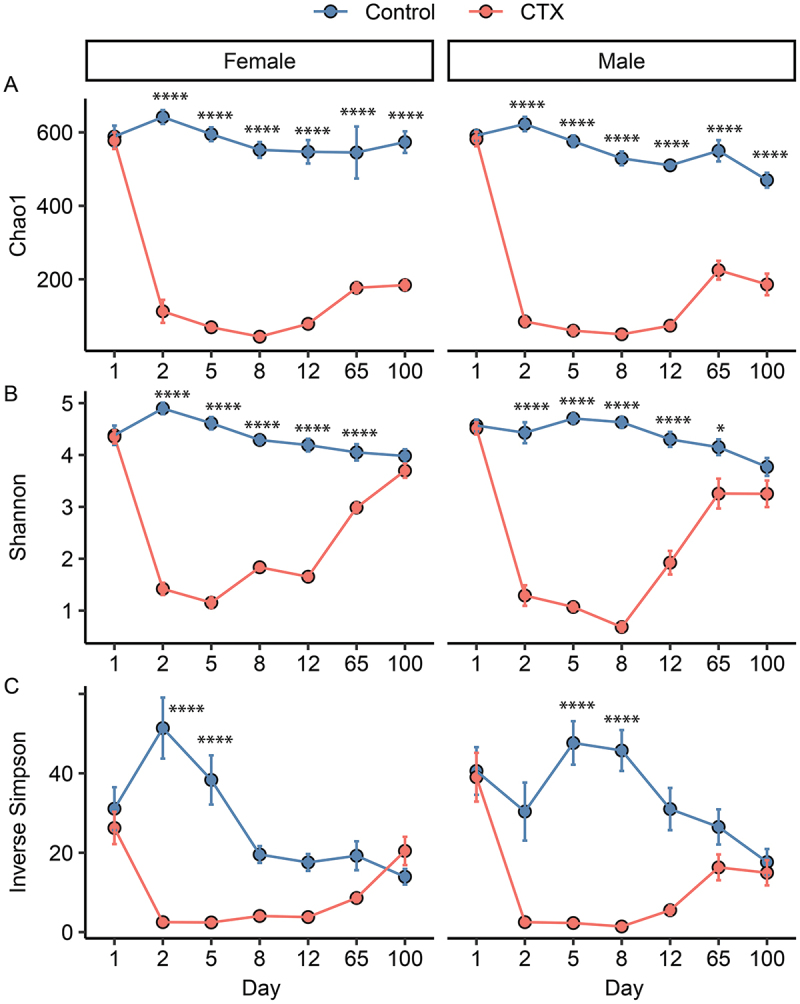
Richness (Chao1, A), evenness (Shannon, B), and heterogeneity (inverse Simpson, C) of 16S rRNA gene profiles of fecal samples from mice administered ceftriaxone (CTX) and time-matched controls. *p < 0.05, ****p < 0.0001.

Three linear mixed-effects models were constructed to assess the effects of Treatment, Day, and Sex on alpha diversity indices in a study involving mice (Figure 3). Chao1 index, an estimator of species richness adjusted for undetected rare species, showed significant interaction effects between Treatment and Day (*F* = 48.98, *p* < 0.001), indicating variability in richness influenced by CTX across different days (Figure 3A). The main effect of Treatment (*F* = 0.19, *p* = 0.66) and Sex (*F* = 0.00, *p* = 0.97) were not significant, suggesting no independent influence of these two factors on Chao1 richness. Shannon index, a measure of species diversity considering both richness and evenness, demonstrated significant interactions between Treatment and Day (*F* = 75.16, *p* < 0.001) and between Treatment, Day, and Sex (*F* = 3.40, *p* = 0.003), highlighting their combined influence on diversity outcomes (Figure 3B). Inverse Simpson index, which assesses the evenness of species abundance, showed significant effects of Treatment (*F* = 8.20, *p* < 0.001) and Day (*F* = 6.38, *p* < 0.001), as well as a significant interaction between Day and Sex (*F* = 3.08, *p* = 0.0069) (Figure 3C). This indicates that the effect of Day on evenness varies by sex, underscoring an interaction effect in shaping community evenness.

Pairwise Tukey’s tests comparing alpha diversity indices between control and CTX-treated mice across different days revealed distinct patterns of recovery dynamics across Chao1 richness, Shannon diversity, and Inverse Simpson evenness. Firstly, Chao1 richness did not show significant recovery in either sex throughout the study period. Both CTX-treated male and female mice consistently exhibited significantly lower richness levels compared to controls from Day 1 up to Day 100, indicating a sustained impact of the intervention on species richness (Figure 3A). Secondly, Shannon diversity displayed a complete recovery for both sexes only by Day 100 in the CTX group, which was not significantly different from controls at this time point. Prior to Day 100, both male and female CTX samples consistently exhibited lower Shannon diversity compared to controls, underscoring a prolonged period needed for diversity metrics to fully normalize following the intervention (Figure 3B). Thirdly, significant sex differences were noted in Inverse Simpson evenness across multiple time points (Figure 3C). For instance, on Day 65, CTX-treated males showed significantly lower evenness compared to CTX-treated females (*p* < 0.05), suggesting a differential response to the intervention between sexes in terms of species evenness (Figure 3C). Similar patterns of sex-specific differences were observed on other days, indicating variability in how males and females responded to the CTX treatment.

**Figure 4.**
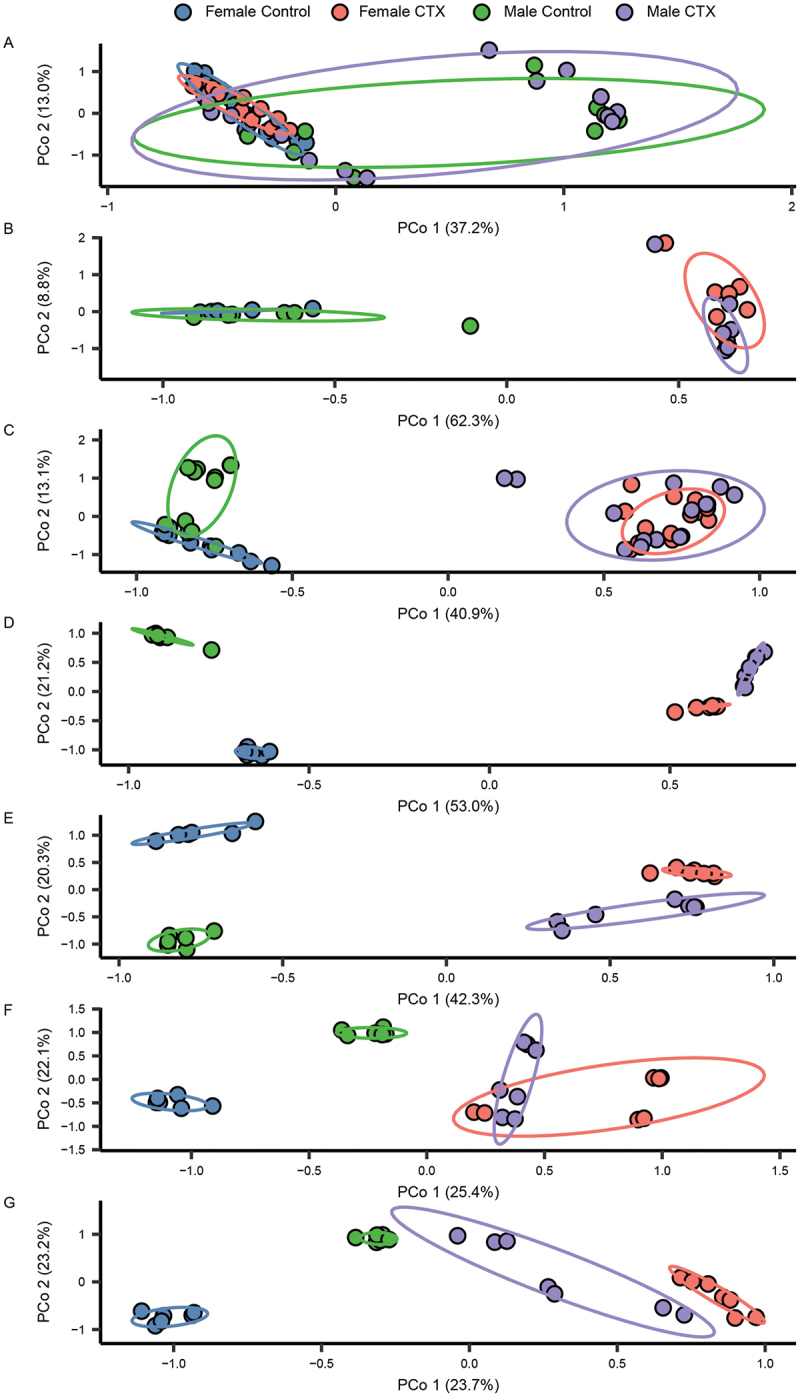
Principal coordinates analysis (PCoA) depicting the variation in structure (Bray-Curtis Index) of fecal 16S rRNA gene profiles from female and male control mice and those administered ceftriaxone (CTX) over time. Panels a-g correspond to samples collected on Day 1, 2, 5, 8, 12, 65, and 100, respectively. CTX was administered 24 hours before sample collection on Day 2 (B) and Day 5 (C).

With respect to beta diversity, there was a high degree of separation between the bacterial profiles of CTX-treated and time-matched controls based on community structure (Figure 4). The PERMANOVA analyses revealed significant shifts in microbial community composition influenced by Treatment, Sex, and their interactions across the study phases. Prior to antibiotic administration on Day 1, Sex significantly shaped microbial community structure (*R*^2^ = 0.18417, *p* = 9.999e-05), and the interaction between Treatment and Sex was non-significant. Following antibiotic treatment from Day 2 to Day 5, there was an evident disruption in microbial structure, indicated by significant effects of Treatment (Day 2: *R*^2^ = 0.59534, *p* = 9.999e-05; Day 5: *R*^2^ = 0.41438, *p* = 9.999e-05), Sex (Day 2: *R*^2^ = 0.04186, *p* = 0.05519; Day 5: *R*^2^ = 0.06663, *p* = 9.999e-05), and their interaction (Day 2: *R*^2^ = 0.03738, *p* = 0.07119; Day 5: *R*^2^ = 0.05355, *p* = 5e-04). This disruption persisted into the recovery phase beginning on Day 8, where Treatment (*R*^2^ = 0.19002, *p* < 0.001), Sex (*R*^2^ = 0.08923, *p* < 0.001), and their interaction (*R*^2^ = 0.05384, *p* = 0.002) continued to significantly influence microbial dynamics. Treatment consistently explained a larger proportion of variation in microbial composition throughout the recovery phase, emphasizing its primary role in driving microbial community structure (Figure 4).

### Effects of CTX on bacterial taxonomic relative abundance

**Figure 5.**
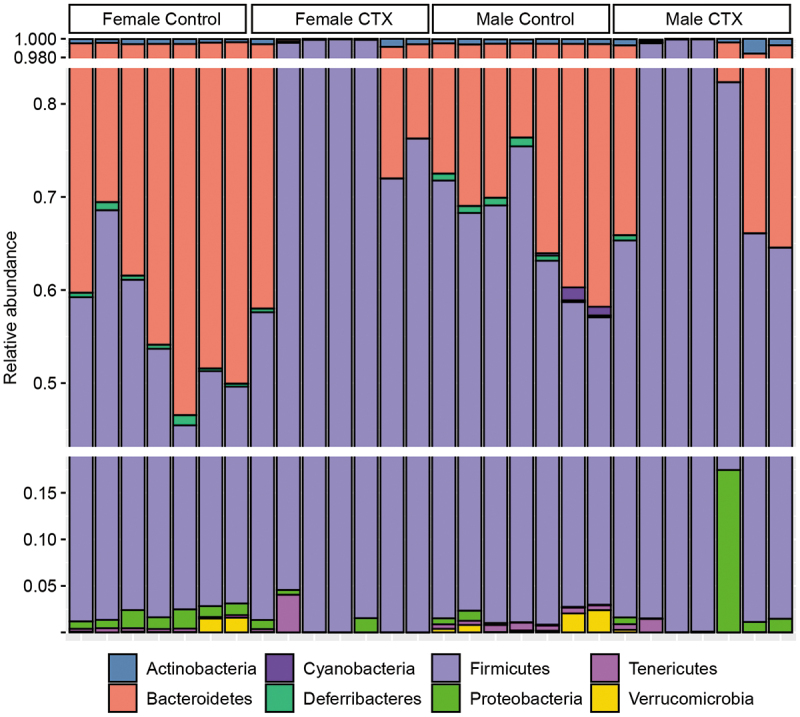
Barplot illustrating the percent relative abundance bacterial phyla among the fecal 16S rRNA gene profiles of female and male control mice and those treated with ceftriaxone (CTX). CTX was administered 24 hours before sample collection on both Day 2 and Day 5.

**Figure 6.**
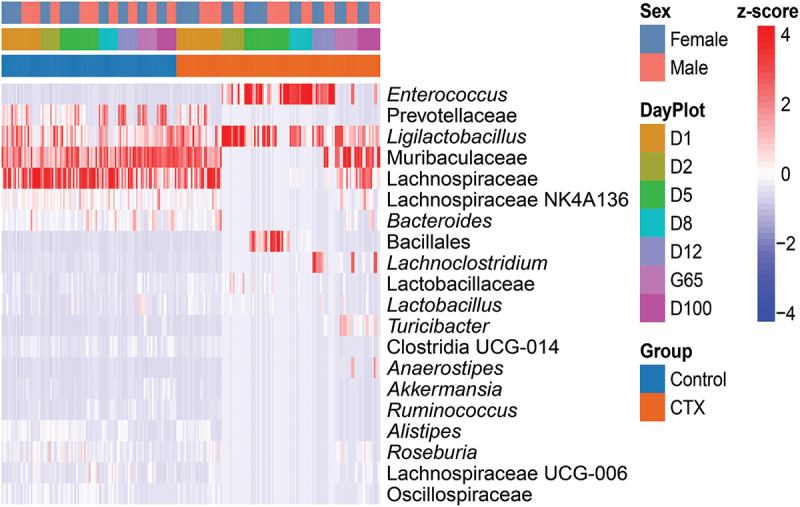
Heatmap illustrating the relative abundance bacterial taxa within the fecal 16S rRNA gene profiles of female and male control mice and those treated with ceftriaxone (CTX). CTX was administered 24 hours before sample collection on both Day 2 and Day 5.

The analysis of microbial taxa abundance in CTX and control samples from females and males during the acute and recovery phases provides insights into how antibiotic treatment affects the gut microbiota of mice. Significant alterations were observed, with specific bacterial taxa showing noteworthy fold differences (FD) (Figures 5–6).

**Figure 7.**
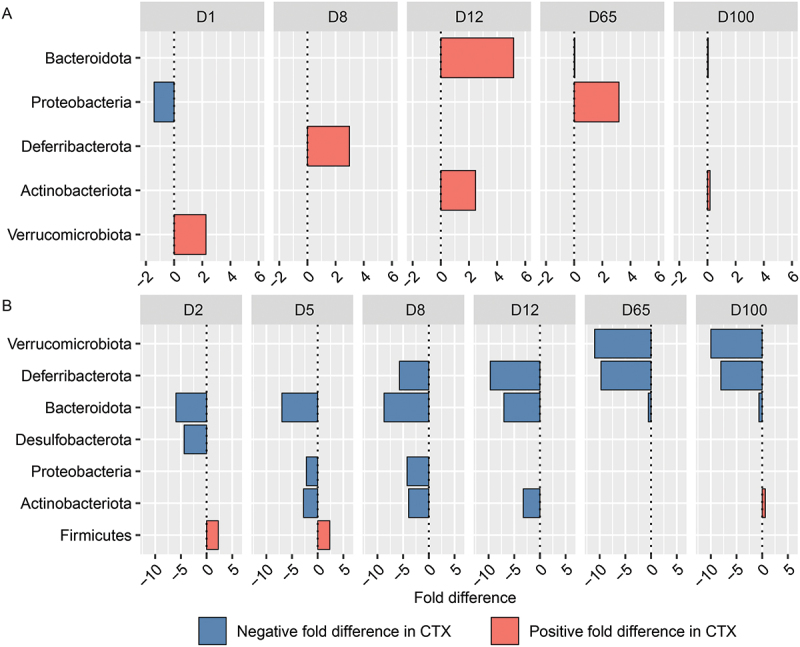
Barplot illustrating the top differentially abundant bacterial phyla selected based on weighted average fold difference across all days, between 16S rRNA gene profiles of fecal samples from (A) male and female mice, as well as (B) control mice and mice administered ceftriaxone (CTX). CTX was administered 24 hours before sample collection on both Day 2 and Day 5.

At the Phylum-level, analysis by sex while controlling for antibiotic treatment group revealed distinct variations in microbial responses during the acute phase of CTX-treated mice time-matched controls. Proteobacteria exhibited significant decreases in males at Day 1 (FD: −1.42, *q* = 0.004), while Verrucomicrobiota showed increases in females on the same day (FD: 2.26, *q* = 0.008). Actinobacteriota significantly increased in males at Day 12 (FD: 2.47, *q* = 0.005), whereas Bacteroidota showed fluctuations at Day 65 (FD: 0.05, *q* = 1.11E–73) and Day 100 (FD: 0.06, *q* = 2.69E–19) in both sexes. These findings indicate sex-specific differences in gut microbiota responses to CTX across various time points (Figure 7A, Table S1).

During the acute phase, while controlling for sex-specific differences, significant shifts in microbial phyla abundances were observed in CTX-treated mice compared to time-matched controls across multiple days. Bacteroidota exhibited marked decreases (FD: −5.97 to −8.72, *q* ≤ 3.18E–20) between Days 2 and 8, alongside reductions in Actinobacteriota (FD: −2.76 to −3.93, *q* ≤ 0.0002) between Days 5 and 8, respectively. Conversely, Firmicutes abundance increased (FD: 2.28 to 2.36, *q* ≤ 8.53E–07) between Days 2 and 5, illustrating the effect of antibiotic treatment on the structure of the gut microbiome (Figure 7B, Table S1).

**Figure 8.**
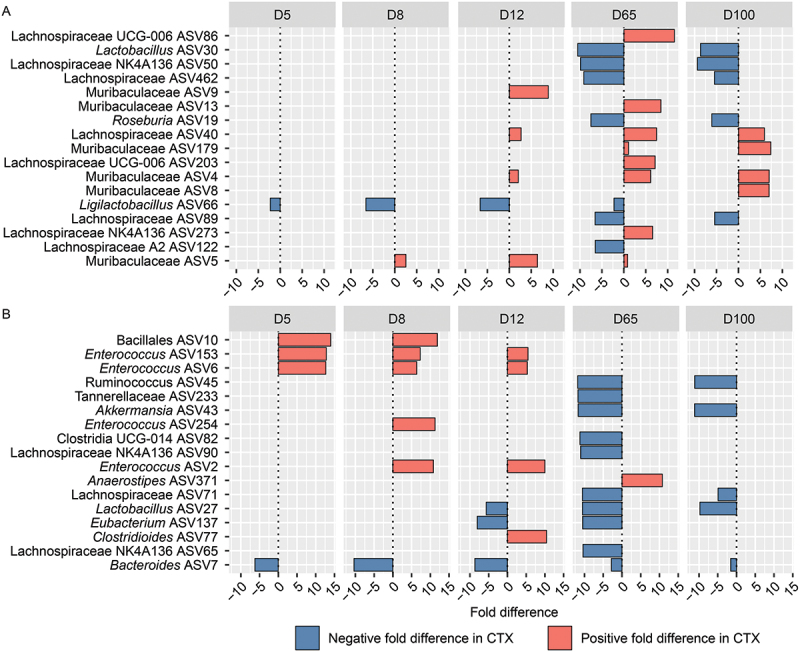
Barplot illustrating the top differentially abundant amplicon sequence variants (ASVs) selected based on weighted average fold difference across all days, between 16S rRNA gene profiles of fecal samples from (A) male and female mice, as well as (B) control mice and mice administered ceftriaxone (CTX). CTX was administered 24 hours before sample collection on both Day 2 and Day 5.

On a finer taxonomic scale, analysis by Sex while controlling for Treatment revealed distinct variations in microbial responses (Figure 8A, Table S2). *Ligilactobacillus*_ASV66 decreased significantly in males at Day 5 (FD: −2.26, *q* = 0.005) and continued to decline markedly by Day 8 (FD: −6.58, *q* = 2.45E–24). Meanwhile, Muribaculaceae_ASV5 increased significantly in females at Day 8 (FD: 2.54, *q* = 0.00018). By Day 12, *Ligilactobacillus*_ASV66 further decreased in males (FD: −6.65, *q* = 1.02E–21), whereas Muribaculaceae_ASV9 (FD: 8.89, *q* = 1.22E–14) and Muribaculaceae_ASV5 (FD: 6.42, *q* = 2.69E–08) showed significant increases in females. Lachnospiraceae_ASV40 also increased in females at Day 12 (FD: 2.68, *q* = 0.005). By Day 65, females exhibited increases in Muribaculaceae_ASV179 (FD: 1.05, *q* = 4.75E–69), while males showed significant decreases in *Roseburia*_ASV19 (FD: −7.51, *q* = 1.09E–21) and *Lactobacillus*_ASV30 (FD: −10.53, *q* = 3.90E–18). Lachnospiraceae_NK4A136_ASV50 also decreased significantly in males at this time point (FD: −9.89, *q* = 8.76E–18). By Day 100, males exhibited decreases in Lachnospiraceae_NK4A136_ASV50 (FD: −9.35, *q* = 3.52E–20) and Roseburia_ASV19 (FD: −6.08, *q* = 6.51E–07), whereas females showed increases in Muribaculaceae_ASV8 (FD: 6.97, *q* = 3.03E–12) and Muribaculaceae_ASV179 (FD: 7.38, *q* = 4.31E–10). These results highlight sex-specific differences in gut microbiota responses to CTX treatment across multiple time points, emphasizing the importance of sex-stratified analyses in microbiome research.

Throughout the study, significant shifts in ASVs were observed in CTX-treated subjects compared to controls, controlling for sex-specific differences (Figure 8B, Table S2). *Enterococcus*_ASV6 (FD: 12.68, *q* = 1.24E–101), Bacillales_ASV10 (FD: 13.99, *q* = 3.75E–63), and *Enterococcus*_ASV153 (FD: 12.82, *q* = 2.37E–42) increased on Day 5, while *Bacteroides*_ASV7 decreased (FD: −6.22, *q* = 4.63E–21). By Day 8, *Enterococcus*_ASV2 (FD: 10.83, *q* = 1.40E–120) and Bacillales_ASV10 (FD: 11.88, *q* = 1.05E–13) increased, and *Bacteroides*_ASV7 decreased (FD: −10.37, *q* = 2.42E–43). Day 12 saw increases in *Clostridioides*_ASV77 (FD: 10.43, *q* = 2.31E–18), and decreases in *Eubacterium*_ASV137 (FD: −8.05, *q* = 8.76E–18) and Lactobacillus_ASV27 (FD: −5.66, *q* = 1.30E–12). By Day 65, *Akkermansia*_ASV43 (FD: −11.70, *q* = 1.14E–58) and *Ruminococcus*_ASV45 (FD: −11.83, *q* = 7.21E–34) decreased significantly, while *Anaerostipes*_ASV371 increased (FD: 10.78, *q* = 2.39E–08), indicating prolonged CTX effects. Day 100 showed similar patterns with *Akkermansia*_ASV43 (FD: −11.20, *q* = 2.76E–38) and Lachnospiraceae_ASV71 (FD: −4.93, *q* = 7.15E–06) decreasing. These findings highlight the dynamic impact of CTX on gut microbiota.

### Analysis of GLT-1 expression in relation to the gut microbiome

**Figure 9.**
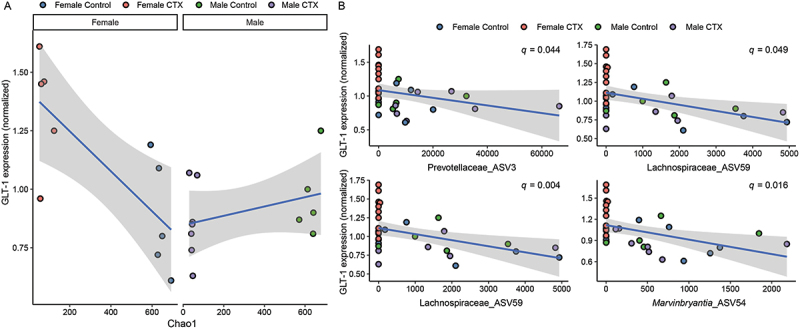
Relationship between gut microbiome characteristics and GLT-1 expression. (A) Regression analyses of Chao1 richness and GLT-1 expression using a generalized linear Model (GLM), demonstrating significant correlations with sex. (B) Negative correlations between specific ASVs and GLT-1 expression identified using Maaslin2.

Analyses of GLT-1 expression relative to alpha diversity metrics indicate that the expression of this transporter is influenced by bacterial richness. For Chao1 diversity, significant effects were observed for Sex (*F* = 4.6050, *p* = 0.04989) and the interaction between Sex and Chao diversity (*F* = 4.7515, *p* = 0.04684), demonstrating that both Sex and its interaction with Chao1 diversity play a role in modulating GLT-1 expression. Additionally, there was a near-significant effect for Chao1 diversity itself (*F* = 4.0162, *p* = 0.06481) and for the interaction between Treatment and Sex (*F* = 3.3475, *p* = 0.08868), suggesting potential influences. Treatment alone did not show significant effects (*F* = 2.7516, *p* = 0.11938) nor did the interaction between Treatment and Chao1 diversity (*F* = 0.8636, *p* = 0.36849), or the three-way interaction of Treatment, Sex, and Chao1 diversity (*F* = 0.3049, *p* = 0.58954). The regressions of Chao1 richness and GLT-1 expression are depicted in Figure 9A.

For both Shannon and Inverse Simpson diversity indices, no effect of diversity, Treatment, or Sex correlated with GLT-1 expression (*p* > 0.05), indicating that neither alpha diversity metric significantly influences GLT-1 expression. Additionally, the combined effects of Treatment, sex, and these diversity indices did not show significant influence on GLT-1 expression. No significant effects were detected for Shannon diversity (Treatment: F = 0.8984, *p* = 0.3593; Sex: F = 0.2179, *p* = 0.6478; Shannon diversity: F = 0.7995, *p* = 0.3863) or the Inverse Simpson diversity index (Treatment: F = 0.5165, *p* = 0.4841; Sex: F = 0.0015, *p* = 0.9693; Inverse Simpson diversity: F = 0.4492, *p* = 0.5136) regarding their influence on GLT-1 expression. Similarly, no significant interactions were observed among Treatment, Sex, and these diversity indices. These results suggest that neither Shannon diversity nor the Inverse Simpson diversity index significantly affects GLT-1 expression in the context of this study.

Further analysis using the Maaslin2 tool revealed specific negative correlations between GLT-1 expression and ASV abundances. Specifically, the abundances of *Marvinbryantia*_ASV54 (coef = −0.8785, *q* = 0.0036), Muribaculaceae_ASV60 (coef = −0.9924, *q* = 0.0156), Prevotellaceae_ASV3 (coef = −0.7893, *q* = 0.0443), and Lachnospiraceae_ASV59 (coef = −1.2747, *q* = 0.0485) were significantly associated with GLT-1 expression (Figure 9B), revealing that all four bacterial taxa were negatively correlated with GLT-1 expression levels.

### Effects of CTX on inferred bacterial functioning

**Figure 10.**
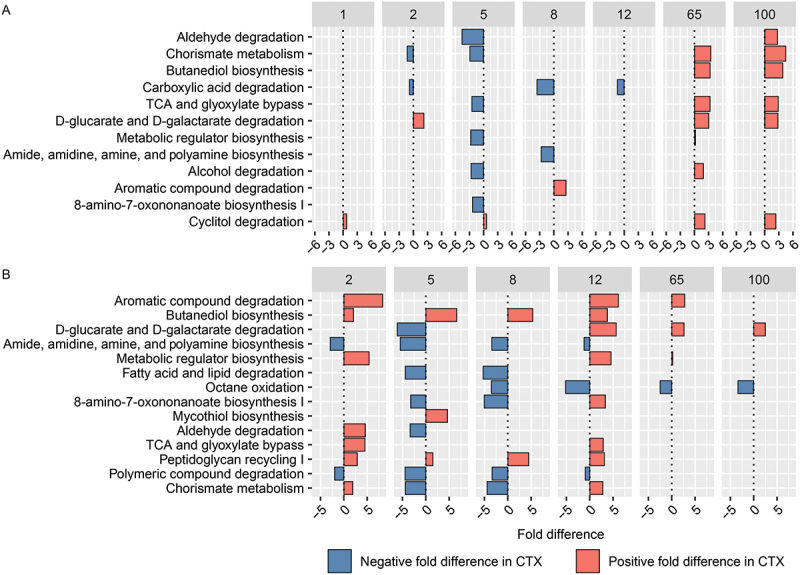
Barplot illustrating the top differentially abundant inferred pathways selected based on weighted average fold difference across all days, between 16S rRNA gene profiles of fecal samples from (A) male and female mice, as well as (B) control mice and mice administered ceftriaxone (CTX). CTX was administered 24 hours before sample collection on both Day 2 and Day 5.

Controlling for Treatment, sex-specific analysis (Figure 10A, Table S3) revealed significant metabolic pathway variations. Cyclitol degradation increased in CTX-treated males on Day 1 (FD: 0.72, *q* = 9.89E–07), while chorismate metabolism decreased substantially (FD: −1.33, *q* = 8.65E–36). Conversely, D-glucarate and D-galactarate degradation increased significantly in females (FD: 2.22, *q* = 0.0075). By Day 5, pathways like aldehyde degradation (FD: −4.59, *q* = 5.91E–05) and alcohol degradation (FD: −2.66, *q* = 0.00029) decreased in males, whereas cyclitol degradation increased (FD: 0.61, *q* = 0). Carboxylic acid degradation decreased in CTX-treated males across multiple days, with further reductions seen on Day 8 (FD: −3.58, *q* = 5.14E–09) and Day 12 (FD: −1.43, *q* = 3.62E–10). In females, pathways including aromatic compound degradation (Day 8: FD: 2.58, *q* = 0.0048) and TCA and glyoxylate bypass (Day 65: FD: 3.32, *q* = 3.08E–06), showed significant increases. By Day 100, cyclitol degradation (FD: 2.36, *q* = 7.41E–12) and butanediol biosynthesis (FD: 3.84, *q* = 3.71E–05) continued to increase in females, highlighting sex-specific responses in gut microbiota metabolic pathways to CTX treatment.

Controlling for sex-specific differences, significant shifts in inferred microbial pathway activity were observed across multiple days (Figure 10B, Table S3). Noteworthy changes included aromatic compound degradation (FD: 8.286547212, *q* = 1.39E–16) and TCA and glyoxylate bypass (FD: 4.506417419, *q* = 1.35E–07) at Day 2, while amide, amidine, amine, and polyamine biosynthesis (FD: −2.923141561, *q* = 1.67E–12) and polymeric compound degradation (FD: −1.981486529, *q* = 7.09E–10) decreased significantly. By Day 5, butanediol biosynthesis showed a substantial increase (FD: 6.617416919, *q* = 2.31E–53), alongside marked reductions in fatty acid and lipid degradation (FD: −4.394145566, *q* = 1.15E–32) and D-glucarate and D-galactarate degradation (FD: −6.100852419, *q* = 1.81E–31). These trends continued through Day 8 with peptidoglycan recycling I (FD: 4.468353378, *q* = 2.03E–80) and butanediol biosynthesis (FD: 5.348720452, *q* = 3.32E–39) showing significant increases, while fatty acid and lipid degradation (FD: −5.288458774, *q* = 1.51E–31) and amide, amidine amine, and polyamine biosynthesis (FD: −3.438353119, *q* = 1.03E–09) decreased substantially. By Day 12, D-glucarate and D-galactarate degradation (FD: 5.664377976, *q* = 9.15E–20) and aromatic compound degradation (FD: 6.113537236, *q* = 1.83E–14) continued to increase significantly. These findings suggest dynamic and profound effects of CTX on metabolic pathways within the gut microbiota.

## Discussion

CTX is a powerful beta-lactam antibiotic, and its use has been adopted in SUD research because of its ability to increase expression of GLT-1 in brain.^1^ Drugs of abuse such as cocaine, nicotine, opioids, and alcohol reduce GLT-1 expression and lead to increased synaptic levels of glutamate in brain areas critical for sustaining drug seeking and relapse.^4^ Glutamate is thought to drive drug seeking and enhance relapse to drug use after extinction.^31^ CTX therefore held promise as a treatment for SUD because its ability to increase GLT-1 would remove excess glutamate from the synapse and reduce seeking and relapse. Initial studies supported this possibility, but more in-depth studies questioned whether CTX exerted its anti-SUD effects via increased GLT-1 expression (see^2^ for review).

By focusing on CTX capacity to enhance GLT-1 expression, SUD studies utilizing this beta-lactam antibiotic have overlooked its potential impact on the gut microbiome. Understanding whether CTX, as administered in SUD studies, induces dysbiosis in the gut microbiome is crucial, given the emerging recognition of the gut microbiome’s significant involvement in the mechanisms of addictive drugs.^2^ Therefore, we investigated the effects of CTX on the gut microbiome using the most commonly prescribed treatment regimen initially demonstrated to enhance GLT-1 expression.^1^ This regimen is also widely employed in SUD studies aimed at mitigating drug seeking and/or relapse.^2^

We confirmed that CTX significantly increases GLT-1 expression, with males showing lower levels compared to females, consistent with studies on sex-specific responses to addictive substances.^32,33^ For instance, estradiol enhances glutamate signaling, potentially boosting GLT-1 expression in astroglia.^32^ Moreover, sex-specific molecular responses to acute and chronic cocaine exposure suggest varied glutamate dynamics,^32^ highlighting potential sex-specific alterations in GLT-1 expression influenced by drugs affecting estrogen-mediated glutamate homeostasis. Additionally, it has been shown that acute and repeated cocaine exposure elicits sex-specific alterations in locomotor responses in rats exposed to chronic social defeat stress, which are potentially mediated by changes in GLT-1 levels. This suggests that the response to cocaine may vary based on the interplay between glutamate dynamics and sex-specific molecular responses.^33^

To assess the gut microbiome’s response to CTX, we initially quantified bacterial load using qPCR. Both male and female mice exhibited a significant reduction in bacterial load by Day 2, with decreases in 16S rRNA gene copies persisting through Day 5. Although bacterial loads approached baseline levels by Day 8, fluctuations were observed on Days 12, 65, and 100, illustrating CTX’s immediate and enduring effects on microbial abundance and the resilience of the gut microbiota.

Our observations align with human gut microbiome studies following antibiotic treatments,^34^ indicating reductions in alpha diversity indices from Day 2 to Day 5, with partial recovery by Day 100. The sustained influence of CTX on species abundance and diversity, as indicated by lower Chao1 index values, contrasts with the gradual normalization of Shannon diversity over time, with no significant sex-based differences observed in these metrics. In contrast, the Inverse Simpson index revealed sex-dependent shifts in taxonomic evenness over time, highlighting distinct responses between male and female mice to CTX treatment in bacterial community evenness. Notably, CTX-treated males displayed significantly lower evenness compared to females, with this difference becoming more pronounced several weeks post-treatment.

Chakraborty et al.^4^ observed significant alterations in bacterial load and alpha diversity using five-week-old male C57BL/6J mice, reporting sustained reductions in microbial load until 6 days after CTX administration, along with continued divergence in Shannon diversity from controls throughout their study (14 days). Conversely, Zeng et al.^6^ using four-week-old male Kunming mice reported significant differences in Chao1 and Shannon diversity 23 d post-CTX treatment compared to controls, though they did not assess immediate effects on gut microbiota. In our study, bacterial load reductions persisted until 6 days post-CTX administration, underscoring CTX’s profound and lasting impact on gut microbiome diversity and structure.

While alpha diversity indices showed partial recovery, beta diversity remained altered, indicating persistent disruptions in microbial community structure. Sex-based differences in baseline microbial profiles were evident even before CTX treatment on Day 1, indicating inherent variations between male and female mice. Our findings align with Chakraborty et al.,^4^ who noted significant shifts in microbial composition during the acute phase using male mice. CTX consistently exerted significant effects on microbial composition throughout the acute and recovery phases. Although sex also influenced microbial community structure across various time points, its impact was relatively modest compared to the pronounced effects of CTX treatment.

The present study employed Maaslin2 to explore correlations between gut microbiota composition and striatal GLT-1 expression, revealing significant negative associations with specific bacterial taxa such as *Marvinbryantia*, Lachnospiraceae Muribaculaceae, and Prevotellaceae and GLT-1 expression. These findings suggest that higher abundances of these taxa are linked to lower GLT-1 expression levels. Given that these bacteria are known producers of short-chain fatty acids (SCFAs),^35–38^ which are products of bacterial fermentation and have been reported to regulate drug seeking in cocaine models,^39^ the findings imply a potential role for gut microbiota-derived SCFAs in modulating GLT-1 expression and its neurobiological implications. SCFAs can influence the modulation of the intestinal barrier,^40^ afferent sensory nerves,^41^ and production of local neurotransmitters,^42^ all of which could contribute to the observed differences in GLT-1 expression and potentially regulate GLT-1 expression.

Subtle differences in the taxa reported in previous studies,^4,43^ and the current study can be attributed to the different primer sets used for amplifying 16S rRNA genes in rodent samples (i.e. V4 vs. V3-V4). Additionally, the different taxonomic assignments reported in all three studies reflect the underlying bioinformatic methodological differences among them, including the use of ASVs vs. operational taxonomic units, and variations in reference bacterial DNA databases (e.g., SILVA database version). These methodological differences can influence the taxonomic resolution of microbial community analyses, leading to subtle differences in taxonomic classifications between studies. With respect to the taxa discrepancies reported in all three studies, the genus *Bacteroides* is within the order Bacteroidales, the genus *Enterococcus* belongs to the family Enterococcaceae, while *Ligilactobacillus* and *Lactobacillus* are both lactobacilli. Therefore, together, the findings of all three studies confirm the intestinal proliferation of enterococci and lactobacilli seen in humans and rodents.^4,44,45^

The current study is the first to investigate the metabolic effect of CTX on the gut microbiome. Antibiotic administration induced significant alterations in microbial pathways with potential implications for SUD and synaptic plasticity.^46,47^ For example, the downregulation observed in amide, amidine, amine, and polyamine biosynthesis pathways after CTX treatment suggests potential changes in the synthesis of biogenic amines crucial for neurotransmission and inflammation modulation,^48^ which may influence susceptibility and response to addictive substances.^49^ Additionally, this downregulation indicates potential changes in neurotransmitter modulation, including histamine and putrescine synthesis,^50^ which may influence synaptic plasticity through the regulation of glutamate receptors, including NMDA receptors, associated with long-term potentiation.^51,52^ Furthermore, the downregulation of fatty acid and lipid degradation pathways induced by CTX indicate potential disruptions in both gut and neuronal membrane integrity, influencing the permeability and transport of addictive substances and their metabolites,^53,54^ as well as alterations in synaptic membrane composition and availability of lipid-derived messengers such as endocannabinoids and prostaglandins, thereby impacting SUD susceptibility and response.^55,56^ Moreover, the observed oscillations in aldehyde degradation pathways may affect detoxification processes, leading to increased oxidative stress and inflammation induced by drugs of abuse. This would further contribute to both the pathophysiology of SUD^57^ and impairment of glutamate receptor function,^58^ exacerbating neurotoxic effects of addictive substances on synaptic plasticity.

Our study reveals significant sex-specific differences in gut microbiota pathway responses to CTX across various time points. Males during the recovery phase (Day 8 to Day 100) consistently exhibited downregulation of carboxylic acid degradation pathways, with fluctuations in pathways such as D-glucarate and D-galactarate degradation. In contrast, females showed dynamic changes, including elevated chorismate metabolism and aldehyde degradation at later time points. During the acute phase (Day 2 and Day 5), males maintained altered metabolic profiles, whereas females demonstrated acute shifts in energy metabolism and detoxification pathways. These findings suggest that sex-specific microbial pathway alterations may regulate synaptic glutamate levels and impact synaptic plasticity differently in response to CTX. This underscores the complexity of microbiome-derived metabolic changes and emphasizes the importance of sex-stratified analyses in microbiome research.

Some limitations of the present study include that GLT-1 expression levels were only measured on Day 5, immediately following the CTX treatment, and that metabolic pathways were inferred based on marker gene sequences (16S rRNA sequencing data). Furthermore, mice treated with drugs of abuse were not used in our study. Future studies employing longer evaluations of GLT-1 levels, metabolomics tools, and the inclusion of mice treated with drugs of abuse to elucidate sex-specific differences in GLT-1 expression and metabolic responses following CTX treatment are warranted. Understanding these sex-specific responses can provide insights into how sex-specific factors influence health outcomes and may inform personalized treatment strategies in the context of CTX treatment.

This study represents the first report of sex-specific impacts of beta-lactam antibiotics on both gut microbiome structure and GLT-1 expression following CTX treatment. The observed variations in bacterial taxa abundance following CTX treatment suggest mechanisms influenced by sex hormones, which play pivotal roles in immune responses and gut microbiota structure. These insights emphasize the critical need to elucidate how sex-specific disruptions induced by antibiotics in gut microbial communities influence synaptic plasticity, addictive behaviors, drug efficacy, and overall health outcomes within the context of SUD. Further exploration of sex as a variable in GLT-1 expression post-treatment and microbiome changes will be crucial for understanding the underlying mechanisms and optimizing SUD therapies.

## Conclusion

While CTX has been explored for its potential in SUD treatment by modulating glutamate levels, the current study reveals that CTX induces profound alterations in the gut microbiome, a factor not previously accounted for in SUD research. These findings highlight the necessity of broadening our understanding of SUD mechanisms to include the gut-brain axis and the influence of microbial communities. Moreover, functional analysis suggests that antibiotic-induced alterations in microbial pathways may have significant implications for synaptic plasticity, neurotransmitter modulation, and inflammatory processes relevant to SUD. Thus, there is a pressing need for further investigation into the complex interplay between gut microbiota, biological sex, synaptic plasticity, and addictive behaviors to identify novel therapeutic targets for addressing SUD effectively.

## Supplementary Material

Table_S3.xlsx

Table_S2.xlsx

Table_S1.xlsx

## Data Availability

The 16S rRNA gene sequencing files for fecal samples have been deposited in the National Center for Biotechnology Information Sequence Read Archive (SRA) as BioProject PRJNA1136882.
